# *N-*docosahexaenoylethanolamine regulates Hedgehog signaling and promotes growth of cortical axons

**DOI:** 10.1242/bio.013425

**Published:** 2015-11-06

**Authors:** Giorgi Kharebava, Mohammad A. Rashid, Ji-Won Lee, Sarmila Sarkar, Karl Kevala, Hee-Yong Kim

**Affiliations:** Laboratory of Molecular Signaling, Division of Intramural Clinical and Biological Research, National Institute on Alcohol Abuse and Alcoholism, National Institutes of Health, Bethesda, MD 20852, USA

**Keywords:** Axon, Docosahexaenoic acid, Sonic hedgehog, Synaptamide, Cyclopamine

## Abstract

Axonogenesis, a process for the establishment of neuron connectivity, is central to brain function. The role of metabolites derived from docosahexaenoic acid (DHA, 22:6n-3) that is specifically enriched in the brain, has not been addressed in axon development. In this study, we tested if synaptamide (*N*-docosahexaenoylethanolamine), an endogenous metabolite of DHA, affects axon growth in cultured cortical neurons. We found that synaptamide increased the average axon length, inhibited GLI family zinc finger 1 (GLI1) transcription and sonic hedgehog (Shh) target gene expression while inducing cAMP elevation. Similar effects were produced by cyclopamine, a regulator of the Shh pathway. Conversely, Shh antagonized elevation of cAMP and blocked synaptamide-mediated increase in axon length. Activation of Shh pathway by a smoothened (SMO) agonist (SAG) or overexpression of SMO did not inhibit axon growth mediated by synaptamide or cyclopamine. Instead, adenylate cyclase inhibitor SQ22536 abolished synaptamide-mediated axon growth indicating requirement of cAMP elevation for this process. Our findings establish that synaptamide promotes axon growth while Shh antagonizes synaptamide-mediated cAMP elevation and axon growth by a SMO-independent, non-canonical pathway.

## Abstract (precis)

**Summary:** Synaptamide, an omega-3 fatty acid metabolite, promotes axon growth while Shh antagonizes synaptamide-mediated axon growth by a SMO-independent, non-canonical pathway.

## INTRODUCTION

Axon development is regulated by precisely timed and spaced interactions between extrinsic cues and intrinsic mechanisms. This process includes axon specification, growth and guidance, which are crucial for establishing proper brain circuitry. The most characterized factors regulating axonogenesis are neurotrophins, such as brain-derived neurotropic factor (BDNF) (Cheng et al., 2011) and an emerging group of morphogens, which include Hedgehogs and bone morphogenetic proteins (BMPs) (Charron et al., 2003; Ji and Jaffrey, 2012). Numerous studies have indicated that many extracellular signals modulate cyclic adenosine monophosphate (cAMP) levels to promote initiation and growth of axons (Cheng et al., 2011; Corredor et al., 2012; Rydel and Greene, 1988; Shelly et al., 2010).

Sonic hedgehog (Shh), a bi-lipidated molecule, functions through a unique signaling mechanism initiated by its binding to the receptor patched-1 (PTCH1). In canonical Hedgehog signaling, this interaction de-represses smoothened (SMO) to activate GLI family zinc finger (GLI)-mediated transcription (Rohatgi et al., 2007). Several non-canonical, Shh-driven pathways have been described, which do not require SMO or are independent of GLI transcription (reviewed by Brennan et al., 2012). During development, Shh signaling through the canonical pathway directs neural cell fate and progenitor proliferation (Wallace, 1999). Shh-dependent axon guidance proceeds independently of GLI and requires activation of Src-family kinases (Yam et al., 2009). In postnatal neurons, Shh expression controls dendrite growth and synapse formation (Harwell et al., 2012; Mitchell et al., 2012). In addition to guiding axons, depending on growth direction, Shh increases or decreases axon length in specific subtypes of retinal ganglion cells (Sanchez-Camacho and Bovolenta, 2008). Although cAMP interacts and antagonizes Hedgehog signaling through protein kinase A (PKA) activation (Niewiadomski et al., 2013), the role of Shh in cAMP regulation or in cAMP-dependent growth of cortical axons is not clear.

A member of the omega-3 polyunsaturated fatty acid family, docosahexaenoic acid (DHA, 22:6n-3) preferentially accumulates in the brain (Diau et al., 2005). Accrual of DHA in the developing human brain peaks during last five weeks of the gestation (Kuipers et al., 2012), thus coinciding with the start of most active axon extension phase that continues after birth (de Graaf-Peters and Hadders-Algra, 2006). Population studies on omega-3 fatty acid deficiency and supplementation, as well as research in animal models indicate a substantial role of these nutrients in brain development and function (reviewed by Fedorova et al., 2007; Innis, 2014; Moriguchi et al., 2013; Ryan et al., 2010). Only a few DHA-derived mediators have been identified to date, including *N*-docosahexaenoylethanolamine, named as synaptamide. This molecule, as opposed to anandamide, the analogous derivative of arachidonic acid (20:4n-6), lacks endocannabinoid activities (Kim and Spector, 2013). Elevation of DHA *in vitro* and *in vivo* stimulates synaptamide biosynthesis (Berger et al., 2001; Rashid et al., 2013; Wood et al., 2010), and synaptamide can be converted to oxygenated products (Yang et al., 2011) or hydrolyzed to DHA by fatty acid amide hydrolase (FAAH) (Bisogno et al., 1999; Wei et al., 2006).

In mouse hippocampal neurons, synaptamide supplementation promotes neurite outgrowth and synaptogenesis (Kim et al., 2011). By elevating PKA/cAMP responsive element binding protein (CREB) signaling, synaptamide enhances neuronal differentiation in cultured neural stem cells (Rashid et al., 2013). Thus, this metabolite recapitulates the previously described neurodevelopmental functions of DHA (Calderon and Kim, 2004; Cao et al., 2009; Katakura et al., 2009). Therefore, in these paradigms, synaptamide is a mediator of the observed activities. Although DHA regulates expression of neurotrophic factors (Ikemoto et al., 2000; Rao et al., 2007), there is no evidence that synaptamide interacts with developmental signaling. Moreover, the role of DHA and synaptamide in the growth of cortical axons has not been addressed.

Hence, the present study was initiated to test if DHA-derived synaptamide regulates the growth of cortical axons and to identify potential interactions of this metabolite with neurodevelopmental and morphogen pathways. Our findings reveal that synaptamide down-regulates Shh signaling, elevates cAMP and similarly to another Hedgehog inhibitor, cyclopamine, promotes extension of cortical axons. We also show that Shh antagonizes the synaptamide-mediated elevation of cAMP and increase in average axon length. These results establish a functional link between conserved Hedgehog signaling and synaptamide that is metabolically formed from a nutritional component DHA.

## RESULTS

### DHA and synaptamide promote axon development in cultured cortical neurons

**Fig. 1.**
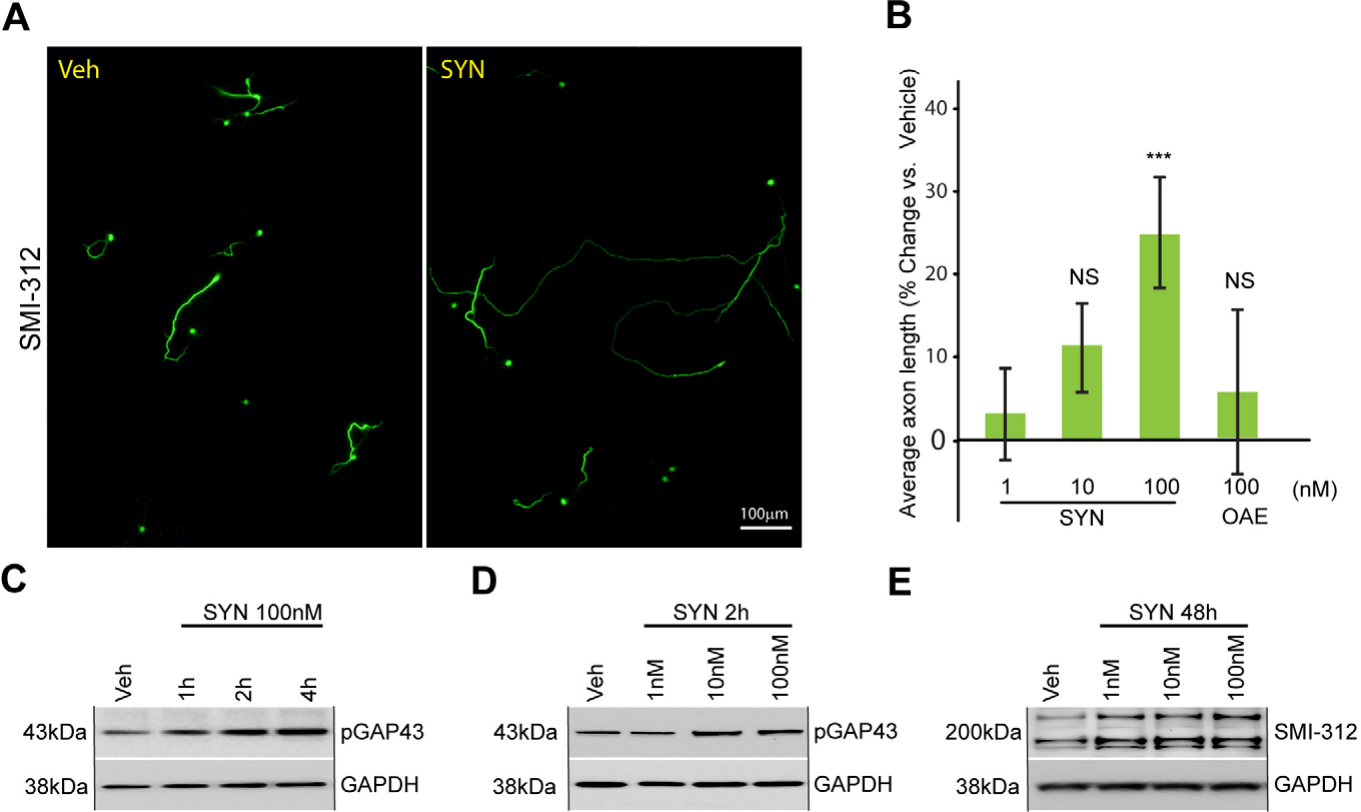
**Synaptamide increases average axon length in cultured cortical neurons.** (A) Representative images of SMI-312-stained cortical neurons supplemented with vehicle and synaptamide. (B) Synaptamide (SYN, 1-100 nM) supplementation for 48 h promotes axon growth, measured as percent average axon length in SMI-312-stained cortical neurons. Synaptamide effect is compared to that of *N*-acylethanolamides of oleic acid (OAE). Results are plotted as percent change in average axon length from vehicle-treated neurons. (C-E) Western blot analysis of cortical neurons supplemented with the indicated concentrations of synaptamide. Equal loading was confirmed by reprobing membranes for GAPDH. (C,D) Synaptamide induced GAP43 phosphorylation as early as 1 h of stimulation. (D) Synaptamide increases GAP43 phosphorylation in a range of 10-100 nM concentrations. (E) Synaptamide elevates phospho-neurofilament (140-220 kDa range, SMI-312) after 48 h of supplementation. In B data represents the average of at least three independent experiments performed in triplicates, error bars are ±s.e.m. At least, 817 cells were analyzed across all the replicates per point. Statistical significance was calculated using one-way ANOVA (*P*=0.0003) followed by Tukey–Kramer post hoc tests; *P<0.05 vs vehicle; ***P<0.001 vs vehicle.

Previous studies have shown that synaptamide, as well as its precursor DHA, promote neurite growth in hippocampal neurons (Calderon and Kim, 2004; Kim et al., 2011). Supplementation of synaptamide at a concentration of 100 nM for 48 h increased average axon length of the cortical neurons by 25% (*P=*0.0001 vs vehicle) (Fig. 1A,B). In the same set of experiments, the ethanolamine derivative of oleic acid (OAE) was not effective in enhancing axon outgrowth (Fig. 1B).

To test if synaptamide can trigger signaling associated with axon development, we evaluated Ser-41 phosphorylation of growth associated protein 43 (GAP43). Synthesis of GAP43 and its phosphorylation particularly at Ser-41 are known to be induced during axon extension for enhanced growth cone activity and guidance (He et al., 1997). Synaptamide increased pSer-41-GAP43 levels as early as 1 h following supplementation (Fig. 1C,D). After 48 h of supplementation, synaptamide at 1-100 nM increased phosphorylation of 160 kDa (medium) and 200 kDa (heavy) subunits of the neurofilaments (Fig. 1E), indicating enhanced activity for axon growth and maturation (Liu et al., 1994).

**Fig. 2.**
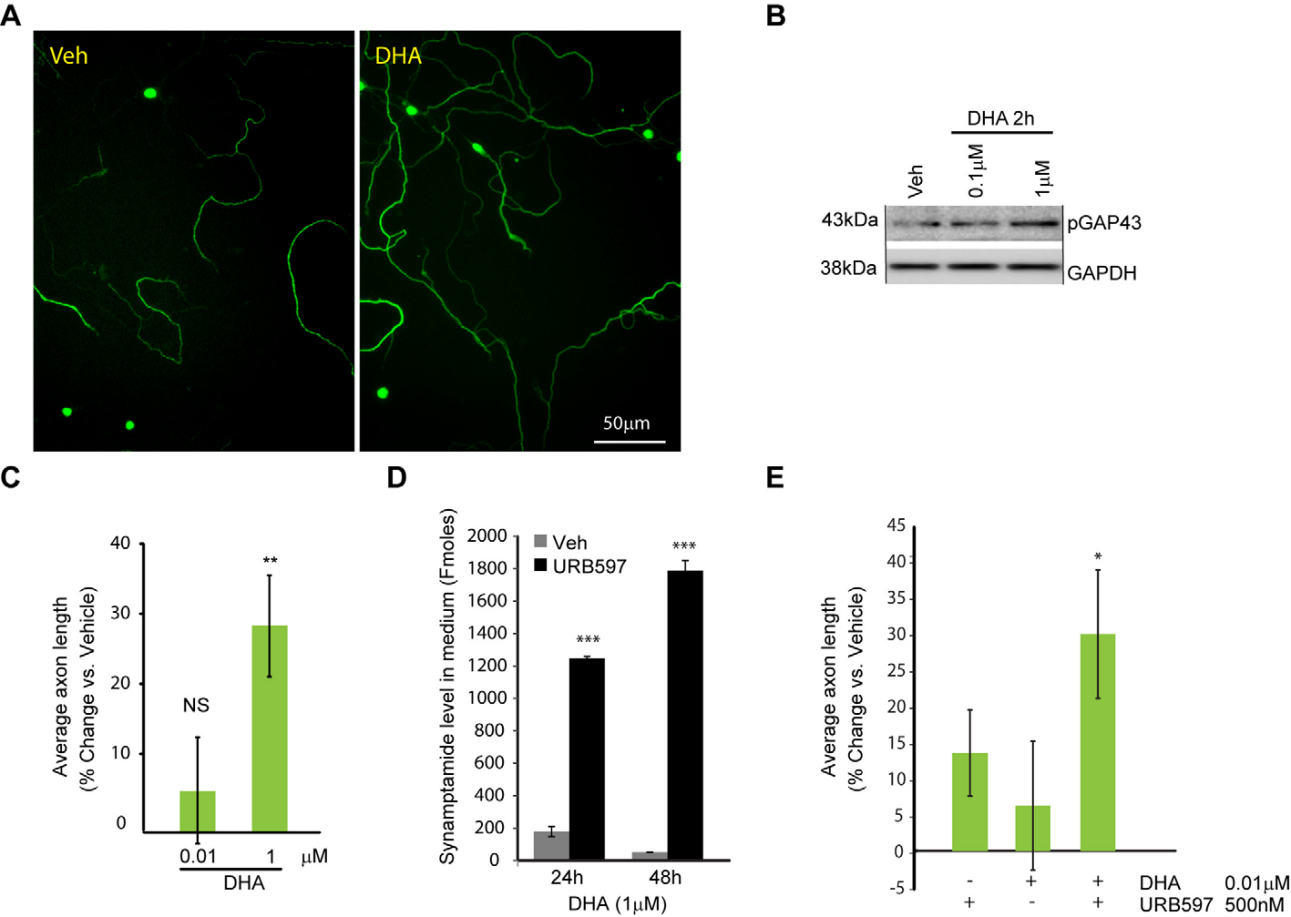
**Docosahexaenoic acid increases average axon length in cortical neurons.** (A) Representative images of the SMI-312-stained cortical neurons from samples treated with DHA, or vehicle. (B) Western blot analysis of the cortical neurons supplemented with indicated concentrations of docosahexaenoic acid (DHA). DHA induced GAP43 phosphorylation within 2 h of stimulation. Equal loading is shown by reprobing membranes for GAPDH. (C) Quantification of average axon length in neurons treated with DHA. Data represents the average of at least three independent experiments performed in triplicates, error bars are ±s.e.m. At least, 682 cells were analyzed across all the replicates per point. Statistical significance was calculated using one-way ANOVA (*P*=0.006) followed by Tukey–Kramer post hoc tests; **P<0.01 vs vehicle. (D) Increased synaptamide levels in response to the FAAH inhibitor URB597. In 24 h after plating, cortical neurons (1×10^6^/ml) were treated with 1 µM DHA or vehicle, in the presence of fatty acid amide hydrolase inhibitor URB597 (0.5 µM). Media were collected 24 or 48 h after treatment, the lipids were extracted and the synaptamide content determined by mass spectrometric analysis. URB597 significantly increased synaptamide levels in the medium of cortical neurons. (E) Quantification of average axon length in neurons treated with sub-optimal concentrations (10 nM) of DHA, with or without URB597. Results are plotted as percent change in average axon length from the vehicle-treated sample. URB597 enhanced the DHA effect on axon development. In D data represent the average of triplicate determination, error bars are ±s.e.m. Statistical significance was calculated by Student's *t*-test, ***P<0.001. In E data represent the average of three independent experiments, error bars are ±s.e.m. At least, 473 cells were analyzed across all the replicates per point. Statistical significance was calculated using one-way ANOVA (*P*=0.03) followed by Tukey–Kramer post hoc tests; *P<0.05 vs vehicle.

Addition of DHA increases synaptamide levels in hippocampal neurons and neural stem cells (Kim et al., 2011; Rashid et al., 2013). Therefore, we tested whether DHA may also affect axon outgrowth in cortical neurons (Fig. 2). We found that supplementation with 1 µM DHA induced GAP43 phosphorylation within 2 h and enhanced cortical axon growth by 28% (*P=*0.006 vs vehicle). Since both DHA and synaptamide increased average axon length, we examined if blocking hydrolysis of synaptamide produced from DHA may affect this process (Fig. 2D-E). Supplementation of 1 µM DHA to cortical neurons enhanced production of synaptamide, peaking around 24 h at about 200 fmol/ml in the media. Co-treatment with FAAH inhibitor, URB597 (500 nM) increased synaptamide levels in the DHA supplemented neuronal media by 7- and 34-fold, at 24 h (*P=*6.3×10^−7^, vehicle vs URB597) and 48 h (*P*=9.8×10^−7^, vehicle vs URB597), respectively (Fig. 2D). Moreover, URB597 was able to enhance axon outgrowth when co-treated with sub-optimal concentrations of the DHA (Fig. 2E). Ten nM DHA was ineffective (6% increase; *P>*0.05) alone but co-treatment with DHA and 500 nM URB597 increased average axon length by 29% above of the vehicle-treated group (*P=*0.03, vs vehicle). These results suggest that axon growth is enhanced by the conversion of the DHA into synaptamide.

### Synaptamide regulates sonic hedgehog signaling in cortical neurons

**Fig. 3.**
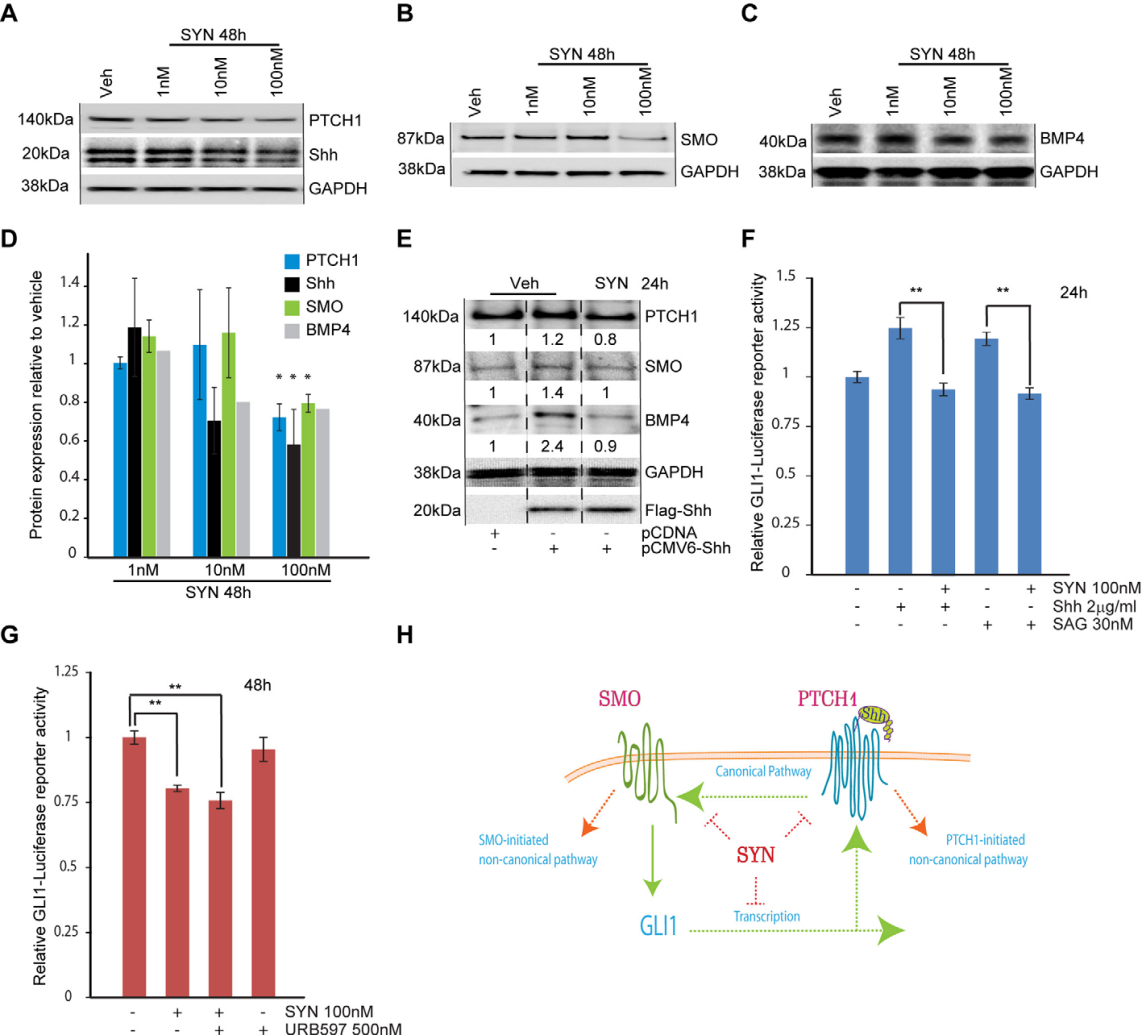
**Synaptamide antagonizes sonic hedgehog signaling in cortical neurons.** (A-C) Representative western blot analysis of the cortical neurons supplemented at *DIV1* with indicated concentrations of synaptamide. Equal loading was evaluated by reprobing membranes for GAPDH. In A, Shh and Patched-1 were probed from the same membrane. In B and C SMO and BMP4 expression levels were evaluated after synaptamide treatment at the indicated concentrations. (D) Quantification of western blots presented in A-C. Error bars are ±s.e.m. Statistical significance for western blots was calculated using Student's *t*-test; *P<0.05 vs vehicle. (E) Synaptamide antagonizes Shh target gene expression in cortical neurons. Immediately after isolation, cortical neurons were electroporated with 0.5 µg pCMV6-Flag-Shh or an equal amount of pCDNA plasmids per 1×10^7^ cells. At 24 h after plating, neurons were treated with 100 nM synaptamide as indicated. After 24 h of treatment, neurons were lysed and analyzed by western blotting for BMP4, PTCH1, Shh and GAPDH, a representative blot of the experiments that were repeated three times is shown. (F,G) Mouse cortical neurons were co-electroporated with a GLI1-luciferase reporter and β-galactosidase expressing plasmids (1 µg pTF-GLI1-Luc-reporter and 0.2 µg EF1aLacZ per 1×10^7^ cells). After plating for 24 h, *DIV1* transfected neurons were treated with synaptamide, Shh (2 µg/ml) or SAG (30 nM) and FAAH inhibitor URB597 (0.5 µM) as indicated. Luciferase activity was evaluated at either 24 or 48 h after treatment. In F and G data represents the average of at least three independent experiments performed in duplicates, error bars are ±s.e.m. Statistical significance was calculated using one-way ANOVA followed by Tukey–Kramer post hoc tests; **P<0.01. (H) Synaptamide antagonizes Hedgehog signaling. Canonical Hedgehog signaling is triggered by transactivation of SMO by Shh-PTCH1 complex. This leads to GLI-mediated transcription and expression of canonical target genes, including PTCH1 itself. Two general types of non-canonical pathways could be designated in Hedgehog signaling. First, SMO- and GLI1-independent signaling initiated by PTCH1 and second, GLI1-independent signaling initiated by SMO. Synaptamide antagonizes Hedgehog signaling by downregulating the expression of key Hedgehog-related proteins, such as PTCH1, SMO and by inhibiting GLI1-mediated transcription.

To understand the potential mechanism of synaptamide-mediated axon growth, a focused screen for genes that are active in neural development was performed, and this effort suggested involvement of sonic hedgehog (Shh) signaling. We found that Shh and its canonical target, the receptor PTCH1 (Chen and Struhl, 1996), were reduced in cortical neurons after 48 h supplementation with 100 nM synaptamide. Shh and PTCH1 levels decreased to 60% and 73% of the vehicle-treated group, respectively (*P=*0.04 and *P=*0.05, respectively) (Fig. 3A-D). Smoothened (SMO), a G-protein coupled receptor (GPCR) and mediator of canonical Hedgehog signaling, decreased to 80% (*P=*0.02) of the vehicle group. The reduction of BMP4, another Shh target gene, was also observed (Fig. 3B-D).

Next, we evaluated whether synaptamide may directly block Shh-induced signaling. DIV0 neurons were transfected with Flag-tagged Shh by electroporation and in 24 h treated with synaptamide for subsequent 24 h. Shh transfection produced strong up-regulation of BMP4 expression and a modest increase in SMO and PTCH1 expression, but these increases were blocked by the treatment with 100 nM synaptamide (Fig. 3E). To test if canonical GLI-mediated Shh activity is affected by synaptamide, DIV0 neurons were transfected with a GLI1-driven luciferase reporter plasmid for 24 h and treated with 2 µg/ml Shh or 30 nM SAG. At 24 h after treatment, Shh and SAG increased GLI transcriptional activity by 24% and 20% respectively, but these increases were completely blocked by synaptamide co-treatment (*P=*0.002 vs Shh alone; *P=*0.004 vs SAG alone) (Fig. 3F). After 48 h of synaptamide treatment, basal GLI1-driven transcription was reduced to 80% of the vehicle group (*P=*1.6E-07) that was not reversed by FAAH inhibitor URB597 (Fig. 3G). Based on these data, we conclude that a DHA metabolite, synaptamide antagonizes Hedgehog signaling by regulating Shh-related gene expression.

### Shh blocks synaptamide-mediated increase in average axon length

**Fig. 4.**
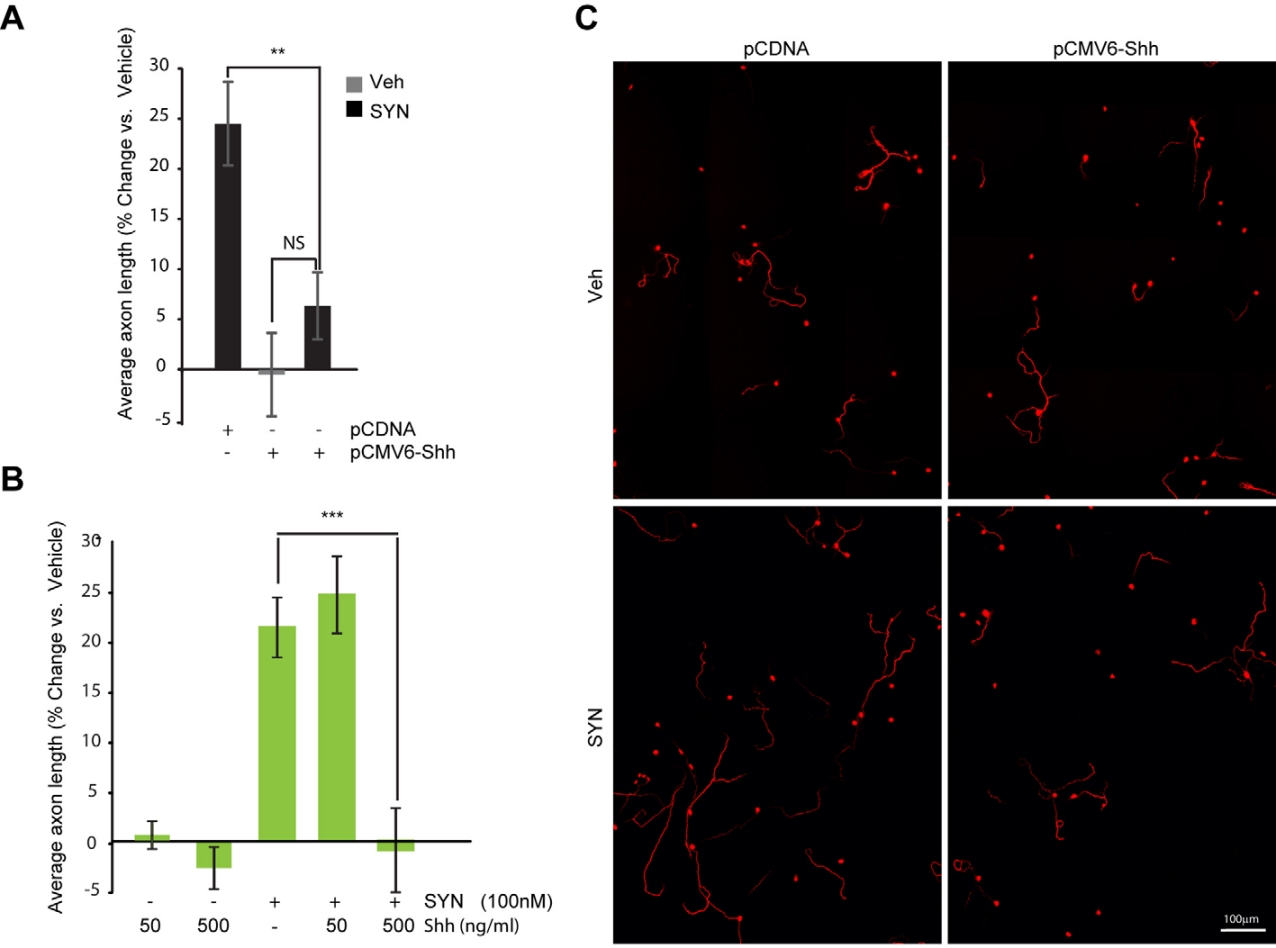
**Sonic hedgehog blocks synaptamide-mediated axon growth in cortical neurons.** (A) Immediately after isolation, cortical neurons were electroporated with 0.5 µg pCMV6-Flag-Shh or equal amount of pCDNA3 plasmids per 1×10^7^ cells. At 24 h after plating, neurons were treated with 100 nM synaptamide for 48 h. Calculation of average axonal length was based on all neurons, regardless of transfection. Results are plotted as percent change in average axon length from vehicle-treated, pCDNA-transfected sample. (B) *DIV1* mouse cortical neurons were supplemented with synaptamide (100 nM) or Shh at 50 ng/ml or 500 ng/ml concentrations. Results are plotted as % change in average axon length from vehicle-treated sample. (C) Representative images of electroporated, SMI-312-stained neurons that were used for quantification. In A,B data represents the average of at least three independent experiments performed in triplicates, error bars are ±s.e.m. At least, 1368 (A) and 905 (B) cells were analyzed across all the replicates per point. Statistical significance was calculated using one-way ANOVA (A, *P*=8.71×10^−7^; B, *P*=7.43×10^−8^) followed by Tukey–Kramer post hoc tests.

We next tested if overexpression of Shh in cortical neurons affects synaptamide-induced axon growth (Fig. 4). Synaptamide supplementation in pCDNA-transfected neurons induced a 25% (*P=*3.8×10^−6^ vs vehicle+pCDNA) increase of average axon length; however, neurons transfected with pCMV6-Flag-Shh showed only an insignificant change compared to control (6% increase, *P=*0.17 vs vehicle+pCDNA) (Fig. 4A). The difference between axon length of synaptamide-treated pCDNA (25%) or Shh-transfected (6%) neurons was significant (*P=*0.002). We also tested whether co-treatment with recombinant Shh will affect the increase in average axon length produced by synaptamide. Cortical neurons were co-treated with 50 or 500 ng/ml of the recombinant Shh. Neither of the recombinant Shh concentrations affected the basal growth of the axons significantly; however, a higher concentration (500 nM) of Shh abolished synaptamide-mediated axon growth completely (Fig. 4B). Specifically, in these experiments synaptamide alone increased average axon length by 21% (*P=*0.003 vs vehicle) and together with 50 ng/ml Shh produced a 25% increase (*P=*0.00002 vs vehicle). Conversely, co-treatment of synaptamide with 500 ng/ml Shh completely blocked the synaptamide-induced increase in axon length (*P=*0.001 vs synaptamide alone) (Fig. 4B).

### Hedgehog regulator cyclopamine promotes axon development

To further understand the role of Shh in inhibiting axon outgrowth, we examined the effect of SMO modulator cyclopamine on axon growth. This compound binds to SMO and regulates its activity and localization (Rohatgi et al., 2009). Cyclopamine also acts as a partial agonist, which similarly to the Hedgehog activator SAG, mediates metabolic reprogramming through SMO (Teperino et al., 2012).

**Fig. 5.**
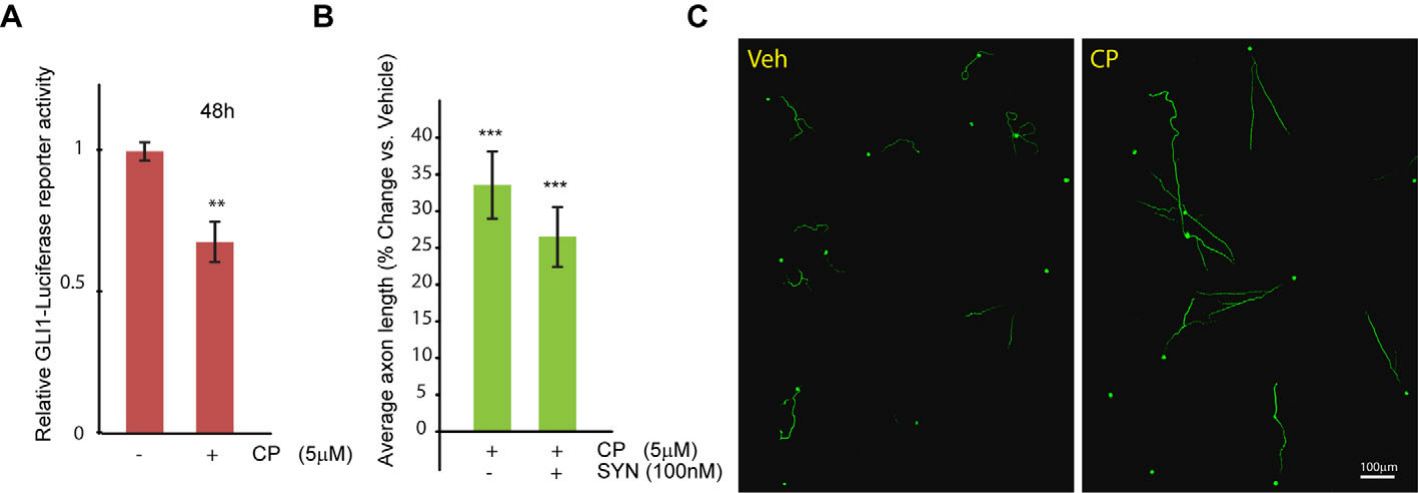
**Cyclopamine inhibits GLI1 transcription and increases average axon length in cortical neurons.** (A) Mouse cortical neurons were co-electroporated with GLI1 luciferase-based reporter and β-galactosidase expressing plasmids (1 µg pTF-GLI1-Luc-reporter and 0.2 µg EF1aLacZ per 1×10^6^ cells). After plating for 24 h, *DIV1* transfected neurons were treated with either cyclopamine (CP, 5 µM) or vehicle, and luciferase activity was evaluated. In B, *DIV1* cortical neurons were treated with cyclopamine (CP), synaptamide or vehicle for 48 h, as indicated. Results are plotted as percent change in average axon length from vehicle-treated sample. At least, 714 cells were analyzed across all the replicates per point. (D) Representative images of the SMI-312-stained neurons treated with vehicle or cyclopamine (5 µM). In A and B data represents the average of at least three independent experiments performed in triplicates, error bars are ±s.e.m. Statistical significance was calculated using one-way ANOVA (A, *P*=0.002; B, *P*=2.28×10^−11^) followed by Tukey–Kramer post hoc tests; ***P<0.001 vs vehicle.

Cyclopamine reduced GLI1-driven transcription (67% of the vehicle; *P=*0.002 vs vehicle), suggesting inhibition of the canonical Hedgehog pathway (Fig. 5A). Furthermore, we found that treatment of cortical neurons with 5 µM cyclopamine produced 34% increase in average axon length in 48 h (*P=*2.2×10^−12^ vs vehicle) (Fig. 5B-C). Interestingly, cyclopamine-treated neurons displayed simplification of axon trajectories pointing to disturbance in the axon guidance mechanisms (Fig. 5C). Co-supplementation of synaptamide and cyclopamine did not result in further significant changes in axon length (*P*=0.26 vs cyclopamine alone) (Fig. 5B).

### Synaptamide and cyclopamine promote axon development independently of SMO signaling

**Fig. 6.**
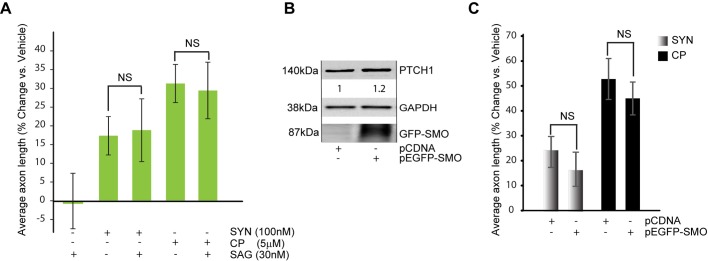
**Synaptamide-mediated axon growth is independent of SMO activation.** (A) *DIV1* cortical neurons were treated with SAG (100 nM) alone or with synaptamide (SYN, 100 nM) and cyclopamine (CP, 5 µM) for 48 h, as indicated. Axonal growth was measured as average axon length in SMI-312 stained neurons. Results are plotted as percent change in average axon length from vehicle treated sample. Data represent the average of at least three independent experiments performed in triplicates, error bars are ±s.e.m. At least, 1149 cells were analyzed across all the replicates per point. Statistical significance was calculated using one-way ANOVA followed by Tukey–Kramer post hoc tests; NS, *P*>0.05. (B) Immediately after isolation, cortical neurons were electroporated with 1 µg pEGFP-SMO or the equal amount of pCDNA. In 24 h after plating, neurons were lysed and analyzed by western blotting. (C) Electroporated neurons from the experiment described in B were separately plated at low density for the axonal analysis and treated with synaptamide (100 nM) or cyclopamine (5 µM). Average axonal length was calculated based on all neurons regardless of transfection. Results are plotted as percent change in average axon length from vehicle-treated, pCDNA-transfected sample. In C data represent the average of at least three independent experiments performed in duplicates, error bars are ±s.e.m. At least, 1423 cells were analyzed across all the replicates per point. Statistical significance was calculated using one-way ANOVA followed by Tukey–Kramer post hoc tests; NS, *P*>0.05.

Both synaptamide and cyclopamine promote axon growth while antagonizing canonical Shh signaling by inhibiting GLI1-driven transcription (Fig. 3F, Fig. 5A). Moreover, Shh which activates SMO (Rohatgi et al., 2007) blocks axon growth produced by synaptamide (Fig. 4). To test if the SMO regulation is required for the observed axon growth by these compounds, we utilized SMO activator SAG that has been shown to stimulate Hedgehog signaling through binding to SMO (Chen et al., 2002). In these experiments synaptamide produced a 17% increase in axon length (*P*=0.003 vs vehicle) and co-treatment with 30 nM SAG produced an 18% increase (*P*=0.01, vs vehicle). Likewise, co-treatment of cyclopamine and SAG produced about a 30% increase that was not different from cyclopamine alone (*P*>0.05) (Fig. 6A).

Next, we asked if the direct increase in SMO levels would reduce synaptamide mediated axon growth. Overexpression of GFP-tagged SMO increased the expression of PTCH1, a Shh target gene (Fig. 6B). Nevertheless, it did not block axon growth promoted by synaptamide or cyclopamine (Fig. 6C). Specifically, pCDNA and pEGFP-SMO expression produced 23% vs 17% (*P*=0.1) increases after synaptamide treatment and 53% vs 45% increases in cyclopamine-treated neurons (*P*=0.5), respectively (Fig. 6C). These results indicate that neither stimulation of SMO nor increase in SMO levels is sufficient for blocking axon growth mediated by synaptamide or cyclopamine.

### Shh blocks synaptamide-mediated increase in cAMP that is required for axon growth

**Fig. 7.**
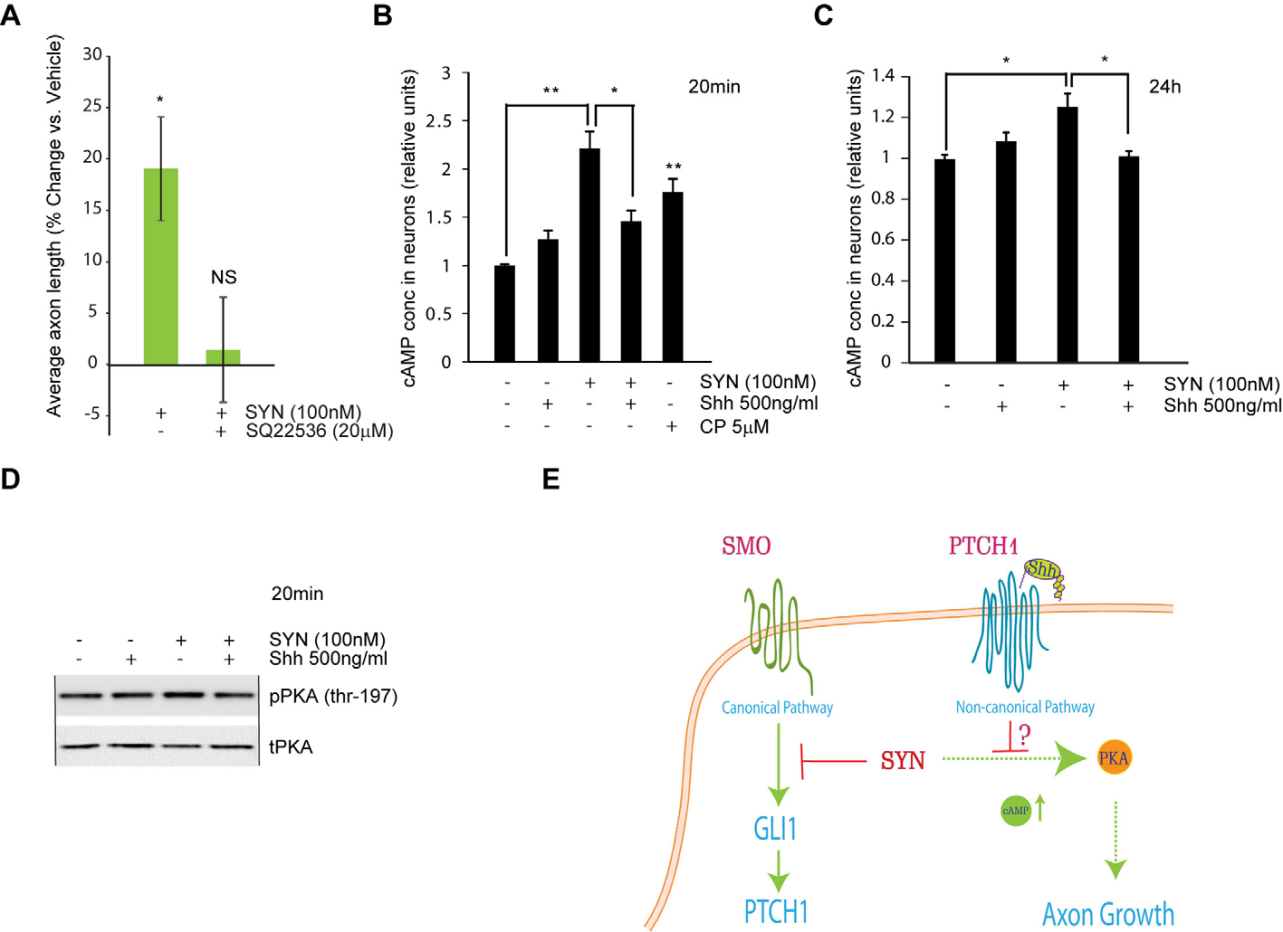
**Shh and synaptamide antagonistically regulate cAMP and axon growth.** (A) *DIV1* cortical neurons were treated with synaptamide (100 nM) alone or with synaptamide (SYN, 100 nM) and SQ22536 (20 µM) for 48 h. Axonal growth was measured as average axon length in SMI-312 stained neurons. Results are plotted as percent change in average axon length from vehicle treated sample. In A, data represent the average of at least three independent experiments performed in duplicates, error bars are ±s.e.m. At least, 596 cells were analyzed across all the replicates per point. Statistical significance was calculated using one-way ANOVA followed by Tukey–Kramer post hoc tests; NS, *P*>0.05. (B,C) Synaptamide increases cAMP and Shh inhibits synaptamide-mediated elevation of cAMP. *DIV1* neurons were treated as shown and at indicated time points cAMP levels in neuron lysates were evaluated using Cyclic AMP XP assay kit (Cell Signaling). In B and C representative experiment from two independent experiments performed in triplicates is shown, error bars are ±s.d. Statistical significance was calculated using Student's *t*-test; *P<0.05, **P<0.001. (D) Western blot analysis of phospho-PKA in the cortical neurons that were supplemented at *DIV1* with indicated combinations of synaptamide (100 nM) and Shh (500 ng/ml). Synaptamide mediates increase in PKA phosphorylation at Thr-197 that is inhibited by Shh. Equal loading was evaluated by reprobing membranes for total PKA. (E) Potential mechanism of synaptamide and Shh regulation of axon development. Synaptamide (SYN) may elevate cAMP and inhibit SMO/GLI1 signaling to facilitate axonal growth. Shh antagonizes synaptamide-mediated increase in average axon length independently of the canonical, SMO-driven pathway.

Since cAMP regulation was proposed as important mediator of axon growth in cortical neurons (Shelly et al., 2010) and synaptamide has been shown to activate PKA/CREB, the downstream signaling of cAMP in neural stem cells (Rashid et al., 2013), we tested if blocking cAMP elevation by SQ22536, a cell permeable adenylyl cyclase inhibitor, will affect the axonal effect of synaptamide. SQ22536, at 20 µM concentration, completely abolished the axon growth by synaptamide (*P*>0.05, synaptamide+SQ22536 vs vehicle) (Fig. 7A). Furthermore, we found that within 20 min of supplementation, synaptamide elevated the cAMP content by more than 2-fold (*P*=0.002) and produced an increase in phosphorylated PKA (Fig. 7B,D). The elevation of cAMP lasted up to 24 h (25% increase, *P*=0.03 vs vehicle) (Fig. 7C). Cyclopamine also elevated cAMP levels at an earlier time point (75% increase, *P*=0.002 vs vehicle) (Fig. 7B). In these experiments Shh reduced the increase in PKA phosphorylation and cAMP at both time points (Fig. 7B-D). Particularly, at 20 min, co-treatment with Shh (500 ng/ml) and synaptamide produced only a 1.5-fold increase in cAMP, significantly less than that produced by synaptamide alone (*P*=0.02). At 24 h, the cAMP level in Shh and synaptamide co-treated neurons was not different from the level in neurons treated with vehicle (*P*>0.05).

## DISCUSSION

In this study, we show that the omega-3 fatty acid ethanolamide synaptamide inhibits Shh signaling, promotes cAMP levels and increases outgrowth of cortical axons. We also demonstrate that the Hedgehog regulator cyclopamine induces a significant increase in average axon length. In addition, we provide evidence that Shh antagonizes axonal effects of the synaptamide through SMO-independent non-canonical signaling, possibly by inhibiting cAMP production.

In the mouse, developing cortical neurons enter the active axon growth phase at embryonic day (E)18, and this continues until around postnatal day (P)7 (Lewis et al., 2013). Thus, to evaluate axon growth, we used P0 mouse pup cortical neurons, which start to extend axons within 24 h of plating in culture. Axon measurements were based on the axon specific marker SMI-312 for highly phosphorylated neurofilaments, which correlate with neuronal maturation (Dahl and Bignami, 1986). SMI-312-detectable epitopes are exclusively localized in axons and perikarya of the neuron (Fig. 1A), providing a tool for simultaneous assessment of the total length of the axons and number of neurons.

We found that synaptamide and DHA significantly increased average axon length in developing neurons (Figs 1,2). We also observed an increase in levels of phosphorylated neurofilaments after synaptamide supplementation and early elevation of phosphorylated GAP43 in DHA and synaptamide treated neurons. Since no inhibitors of DHA to synaptamide conversion have been described, we used FAAH inhibitor URB597 to block hydrolysis of synaptamide into DHA and evaluate which of these compounds is primarily responsible for axon growth. We found that URB597 increased synaptamide level and promoted axon growth by sub-optimal concentrations of DHA (Fig. 2D-E). These results suggested that the production of synaptamide by neurons mediates the effect of the DHA on axon development. Thus, we provide the first evidence that synaptamide, an endogenous metabolite of DHA, a nutritional omega-3 fatty acid, stimulates axonogenesis. Our study extends and agrees with the previous reports on neuritogenic role of DHA and synaptamide in hippocampal neurons. In those studies, microtubule-associated protein 2 (MAP2)-stained hippocampal neurons were found to extend dendrites more efficiently if supplemented by 1 µM DHA or 10-100 nM synaptamide (Cao et al., 2009; Kim et al., 2011). These findings provide cellular and molecular evidence to support previous proposals that omega-3 metabolites have an essential function in brain development (Carlson and Salem, 1991; Innis, 2014).

Our results indicated that synaptamide supplementation reduces expression of Shh as well as Shh receptors and gene targets including PTCH1, SMO and BMP4 in cortical neurons (Fig. 2A). Synaptamide antagonized the Shh overexpression-induced elevation of BMP4 and inhibited GLI1 activity induced by the Shh treatment for 24 h, while reducing basal GLI1-dependent transcription after 48 h of treatment. These data indicated that synaptamide inhibits canonical Hedgehog signaling (Fig. 3H). Shh-regulated BMP4 levels are only partially dependent on SMO signaling, indicating involvement of non-canonical pathway in this target gene expression (Astorga and Carlsson, 2007).

Inhibition of the Hedgehog pathway by synaptamide may employ several potential mechanisms described previously. In neural stem cells, synaptamide elevates PKA/CREB signaling (Rashid et al., 2013). It is likely that the cAMP/PKA-dependent mechanism, which is antagonistic to Shh at multiple levels (Pan et al., 2006; Yue et al., 2009), may contribute to synaptamide inhibition of the Hedgehog pathway. The role of small lipid mediators in PTCH1-dependent SMO regulation has been proposed earlier (Myers et al., 2013; Taipale et al., 2002). Recently, polyunsaturated fatty acid ethanolamides, that are structurally similar to synaptamide, have been shown to bind to and inhibit SMO (Khaliullina et al., 2015). Therefore, we cannot rule out direct modulation of SMO, PTCH1 or other Shh related targets by synaptamide. Although direct molecular targets of synaptamide remain obscure, our findings reveal the novel functional connection between this omega-3 metabolite and Shh, a multifunctional morphogen that is critical for development.

We showed that Shh treatment and overexpression blocked the increase in average axon length mediated by synaptamide in cultured cortical neurons (Fig. 4). The sensitivity of the synaptamide effect to Shh directly implicates involvement of the Hedgehog pathway in axon length regulation. Moreover, we found that the Hedgehog regulator cyclopamine was effective in triggering cAMP elevation (Fig. 7B) and increasing the average axon length of cortical neurons (Fig. 5). These findings are in agreement with previous reports that demonstrated the role of Shh in axon development (Harwell et al., 2012; Yam et al., 2009). Although axon guidance is a most studied aspect of Shh function in the developing nervous system, sparse information exists on the function of Hedgehog signaling in the direct regulation of axon length. In a recent study, Zic2-positive and Isl2-positive types of retinal ganglion cells (RGC) that project axons to ipsilateral and contralateral directions from developing retina, exhibited a differential axon growth response to Shh. When respective explants were examined *in vitro*, Shh inhibited the growth of axons in ipsilateral projecting RGCs but stimulated in contralateral projecting RGCs (Sanchez-Camacho and Bovolenta, 2008). Thus, Shh displays the ability to regulate axon length in a developmental context-dependent manner.

Currently, three principal signaling pathways are proposed downstream of Shh, which mediate pleiotropic effects of this molecule; a canonical signaling that requires SMO-dependent activation of GLI1 transcription, and non-canonical pathways that can be either SMO-dependent or PTCH1-dependent. The canonical pathway is involved in neural cell fate determination (Dessaud et al., 2008) while the SMO-dependent but transcription-independent non-canonical pathway that involves Src-family kinases has been implicated in axon guidance (Yam et al., 2009).

Synaptamide which downregulated the SMO-dependent branch of the Hedgehog signaling, and cyclopamine which is known to inhibit SMO, increased average axon length in cultured cortical neurons. Both synaptamide and cyclopamine elevated cAMP and inhibited GLI1-driven transcription indicating inhibition of canonical Hedgehog signaling by these compounds. Although cAMP is well-established to antagonize Hedgehog signaling, emerging data indicate that SMO inhibition can also contribute to cAMP elevation by reducing G_i_ signaling (Shen et al., 2013). Apart from canonical Hedgehog signaling, cyclopamine was also shown to regulate a SMO-dependent non-canonical pathway. Concurrent to SMO/GLI1 signaling inhibition, this drug activates the SMO/Ca2+/AMP-activated protein kinase (AMPK) axis in adipocytes, establishing a Warburg-like glycolytic state (Teperino et al., 2012). Therefore, cyclopamine and synaptamide may function through either inhibition of the canonical signaling or regulation of SMO-dependent non-canonical branches to promote the growth of cortical axons. Nevertheless, we show that 30 nM SAG, the compound that competes with cyclopamine for SMO binding and activates Hedgehog signaling through binding to SMO, failed to block the synaptamide- or cyclopamine-induced increase in cortical axon length. Moreover, overexpression of GFP-tagged SMO stimulated expression of PTCH1, a canonical Hedgehog target, but was ineffective in blocking axon growth induced by synaptamide in cortical neurons. Although Shh activates SMO and synaptamide inhibits the same pathway downregulating PTCH1 and Shh, our results suggest that Shh regulation of synaptamide-mediated axon growth does not require activation of canonical Hedgehog signaling. This conclusion is also supported by fact that GLI1 is expressed mostly in astrocytes rather than neurons in the mature cortex (Garcia et al., 2010), indicating a less important role of canonical pathway for the Shh functions in neurons.

Several non-canonical pathways have been described for Hedgehog signaling in various experimental models. SMO-dependent, but GLI1-independent signaling downstream of Shh triggers calcium transients in spinal cord neurons (Belgacem and Borodinsky, 2011), or activates small GTPases in endothelial cells (Chinchilla et al., 2010; Pathi et al., 2001; Polizio et al., 2011). Interestingly, cytoskeletal rearrangements necessary for lamellipodia formation and migration in mesenchymal fibroblasts required omega-6 polyunsaturated fatty acid metabolites, but not GLI1 activation (Bijlsma et al., 2007). Shh signaling, driven exclusively by PTCH1 that is SMO/GLI1-independent, includes induction of apoptosis in endothelial and neuroepithelial cells (Chinchilla et al., 2010) and regulation of the Cyclin B1/CDK1 pathway in basal cell carcinoma (Wang et al., 2011).

The blocking of synaptamide-mediated axon growth by the adenylyl cyclase inhibitor, SQ22536, strongly suggests a requirement of cAMP signaling for this effect (Fig. 7A). We show that synaptamide-induced cAMP production (Fig. 7B,C) (Kim and Spector, 2013; Rashid et al., 2013) is reduced by Shh treatment, highlighting potential mechanism for observed Shh and synaptamide antagonism in axon growth. We cannot completely exclude modulation of cAMP levels by synaptamide and cyclopamine through G-proteins downstream of SMO (Shen et al., 2013). Nevertheless, insensitivity of synaptamide-mediated axon growth to SMO manipulations (Fig. 6) indicate that additional, non-canonical Shh signaling may antagonize the synaptamide effect. A recent study demonstrated that GPR161, a GPCR that co-localizes with PTCH1/SMO in an active sub-compartment, inhibits Shh signaling through cAMP/PKA elevation and GLI repression (Mukhopadhyay et al., 2013). Elevation of cAMP was shown to trigger axonal polarization and growth in cortical neurons (Shelly et al., 2010), and suppress GLI1 transcription by SMO-independent mechanisms (Yue et al., 2009). Hence, we propose that axon growth is triggered by synaptamide interaction with a cAMP-elevating target, which is Shh-sensitive, in SMO-independent manner as illustrated in the summarizing schematic in Fig. 7E.

## MATERIALS AND METHODS

### Antibodies, constructs and other materials

The antibodies were obtained from following sources: mouse anti-SMI-312 (Covance), rabbit anti-phospho-GAP43 (Ser-41; Sigma), rabbit anti-GAPDH, rabbit anti-sonic hedgehog, rabbit anti-phospho-PKAc (thr197), rabbit anti-PKAc (Cell Signaling), mouse anti-patched-1 (H-267, Santa-Cruz), rabbit anti-BMP4 (Abcam), rabbit anti-smoothened (Origene), goat anti-mouse Alexa Fluor 488 and 555 (Life Technologies). Empty construct pCDNA3.1 and EF1αLacZ for the expression of β-galactosidase have been described previously (Kharebava et al., 2008). Myc and FLAG-tagged human Shh expression construct pCMV6-Entry-myc-Flag-Shh from Origene. Vectors expressing mouse smoothened protein pEGFP-mSmo (Chen et al., 2002) is from Addgene (Plasmid 25395). For GLI1 transcriptional assays, the pGLI1-Luc Reporter plasmid was used (Signosis). Luciferase assay reagents and β-galactosidase quantification kits were from Promega. Cyclic AMP XP assay kit for the cAMP level quantification was from Cell Signaling Technology. Recombinant murine Shh was from Peprotech. Docosahexaenoic and other polyunsaturated fatty acids, essentially fatty acid-free BSA, SQ22536, and α-tocopherol were obtained from Sigma, and URB597 and cyclopamine were from Tocris; SAG was from Calbiochem.

### Synaptamide preparation and drug treatments

*N*-acylamine derivatives of polyunsaturated fatty acids were prepared and analyzed as described in (Kim et al., 2011). Briefly, methylene chloride solutions of fatty acids were mixed and allowed to react with d_0_- or d_4_-ethanolamine. The reaction product was washed and analyzed for purity by using ESI/MS and gas chromatography. Lipids were extracted, separated on BDS Hypersyl C_18_ column and quantified using an Agilent 1290 UHPLC coupled to TSQ Quantum Ultra mass spectrometer (Thermo Scientific) for analysis of synaptamide content in neuronal culture media. The mass spectrometer was in the positive ion multiple reaction monitoring (MRM) mode and d_4_-synaptamide was used as an internal standard.

Polyunsaturated fatty acid and their *N*-acylethanolamine working solutions (100×) were made under an argon environment in amber glass bottles using 0.5%BSA (essentially fatty acid-free) and 40 μM α-tocopherol in Neurobasal media as a vehicle. URB597, SAG, and cyclopamine were dissolved in dimethyl sulfoxide (at 10 mM) and for treatments diluted to 500× working solutions in neurobasal media. Drug co-treatments were performed 30 min before synaptamide supplementations. Recombinant Shh was in 0.05%BSA/PBS at 5 µg/ml stock solution and was applied as indicated. All treatments, unless stated otherwise, were performed at DIV1.

### Western blotting and immunofluorescence

For western blotting analysis, conventional SDS-PAGE and immunoblotting techniques were used. Briefly protein lysates were prepared in RIPA lysis buffer containing: 50 mM Tris 1% NP40, 1% SDS, 0.25% sodium deoxycholate and protease and phosphatase inhibitor cocktails (Sigma). SDS-PAGE separation was performed using the Life Technologies Novex NuPAGE SDS-PAGE gel system and transfer was performed using the Bio-Rad Trans-Blot Turbo blotting apparatus.

For immunocytochemistry, neurons were fixed in 4% PFA, washed three times and incubated with primary antibody for 4 h at room temperature. SMI-312 antibody (1:1000) was applied in 0.3% Triton X-100, 5% BSA, PBS solution after an additional permeabilization step with 0.5% NP40/PBS solution for 15 min. After washing samples three times with 0.1% Triton X-100/PBS solution, secondary Alexa Fluor conjugated antibody (1:500) was applied for 30 min. Stained cells were stabilized using 80% glycerol solution.

### Luciferase gene reporter and cAMP assay

For the GLI1-Luciferase assay, neurons were electroporated immediately after isolation using Nucleofector 4D. Transfection with pGLI1-Luc reporter plasmid (Signosis) and EF1αLacZ for the expression of β-galactosidase (10:1 ratio, total of 1 µg plasmid/1×10^7^ neurons/cuvette) was followed by plating the cells at 1×10^3^ cells/cm^2^ density, in PDL-coated 24-well plates. At *DIV1,* transfected neurons were treated for 24 or 48 h. After treatments the transfected neurons were lysed in Luciferase assay buffer (Promega). Aliquots of the lysates were used to quantify chemiluminescence by luminometer and β-galactosidase by spectrophotometer. Luciferase activity was normalized using β-galactosidase expression results, and final reporter activities are presented as changes relative to the vehicle-treated sample.

Cyclic AMP XP assay kit (Cell Signaling) were used to measure cAMP levels. In these experiments, neurons were plated with 1×10^3^ cells/cm^2^ density in Poly-D-lysine (PDL)-coated 24-well plates. After a 1 h starvation period, the cells placed back in regular media and were stimulated with synaptamide and Shh for the indicated time points. Experiments were stopped by aspirating the medium, and lysing cells in 100 μl of lysis buffer. In lysates, cAMP concentrations were measured according to manufacturer's instruction. Results were presented as relative to the control values.

### Cortical neuron cultures and nucleofection

Mouse cortical neuron cultures were prepared from *P0* C57BL/6N pups as described in (Habas et al., 2006) with some modifications. Briefly, pup cortices were dissected, digested with papain and triturated before diluting into Neurobasal media containing Glutamax and B27 supplements (Life Technologies). Neurons for axon outgrowth studies were plated at a density of 1×10^2^ cells/cm^2^ and for western blotting analysis 1×10^3^ cells/cm^2^. Neurons were plated on PDL (Sigma) coated 24-well plates or 60 mm culture dishes. All experiments involving animals were carried out in accordance with the guiding principles for the care and use of animals approved by the National Institute on Alcohol Abuse and Alcoholism (LMS-HK31).

For the experiments involving plasmid transfections, neurons immediately after isolation were electroporated using the 4D Nucleofector system from Lonza. Plasmid and Nucleofector reagent mixes and all procedures were prepared and carried out according to the manufacturer's instructions. Briefly, 1×10^7^ neurons and 0.5-1.5 µg plasmid was used per electroporation cuvette. After treatment, the neurons were diluted and plated with densities similar to the regular culture. According to the manufacturer, as well as based on our evaluation, transfection efficiency achieved by this method was around 50-70% of total surviving neurons. Neurons obtained from the same electroporation cuvette were used for both axon quantification and western blotting analysis.

### Axon length quantification and statistical analysis

Axon length was quantified by automatic tracing using the neurite outgrowth module of Metamorph (Molecular Devices). In this module, SMI-312 stained neuron images that display exclusive signal from axons and perinuclear area were evaluated. The signal from the perinuclear area provided the body count and the signal from axons provided total axon length per image. About 15 random images (10× magnification) were collected for each replicate for axon length calculation. Every test included at least triplicates per treatment group, and each image contained several neuronal bodies. Final data represented the cumulative result from at least three independent experiments, derived from three different cultures. Selected image sample size was based on necessary statistical power calculations for confident identification of the axon growth in synaptamide-treated neurons (Power>0.8). Morphometric data obtained by automatic tracing by the neurite outgrowth module were logged in the Excel (Microsoft) spreadsheet. After automatic tracing, each image was manually inspected for potential software mistakes in tracing or neuronal body count and corrected as necessary. Actual average axon length measurements for each image within the experiment were transformed into percent average axon length that was relative to the mean axon length in the respective vehicle-treated group. Therefore, the final representation of the data is percent change from the average axon length of the vehicle/control group.

Statistical analysis was performed using Statistician V2, MS Excel add-in software. Significance quantifications are by one-way analysis of variance (ANOVA) followed by Tukey–Kramer pairwise tests.
